# Left Ventricular Noncompaction Cardiomyopathy in an Elderly Patient: A Case Report and Literature Review

**DOI:** 10.7759/cureus.38305

**Published:** 2023-04-29

**Authors:** Tetyana Okan, Homayoon Lodeen, Michael Abawkaw, Taras Stetsiv, Volodymyr Semeniv

**Affiliations:** 1 Department of Internal Medicine, Lviv National Medical University, Lviv, UKR; 2 Department of Internal Medicine, Jamaica Hospital Medical Center, New York, USA; 3 Department of Radiology, St. Paraskeva Medical Center, Lviv, UKR; 4 Department of Cardiology, St. Paraskeva Medical Center, Lviv, UKR

**Keywords:** noncompaction cardiomyopathy, mri cardiac, systolic dysfunction, myocardial trabeculations, echocardiography, heart failure, genetic cardiomyopathy, cardiomyopathy, spongy myocardium, left ventricular noncompaction

## Abstract

Isolated left ventricular noncompaction cardiomyopathy (LVNC), also known as spongy myocardium, is an extremely rare congenital disorder belonging to unclassified cardiomyopathies by the World Health Organization and classified as a genetic cardiomyopathy by the American Heart Association. Adult prevalence is 0.017-0.26% in observational echocardiographic studies. The disease occurs due to the intrauterine arrest of normal myocardial compaction, leading to left ventricular dysfunction. Reported mortality is high, ranging from 35 to 47% over a 42- to 72-month follow-up period. Knowledge regarding proper diagnosis, morbidity, and prognosis is limited; thus, this disease is subdiagnosed. Our aim is to highlight a diagnostic approach to LVNC in an elderly patient and to stress specific diagnostic signs that make the disease more recognizable. We are reporting a case of noncompaction cardiomyopathy in a 62-year-old male without any significant past medical history who was referred to our clinic for arrhythmia evaluation. The patient had several brief episodes of palpitations over the past two months. On physical examination, he presented a blowing systolic murmur at the apex and an irregularly irregular rhythm. The 12-lead electrocardiogram (ECG) demonstrated atrial fibrillation and ST-T segment depression in the V4-V6 leads. A transthoracic echocardiogram (TTE) showed signs of dilated cardiomyopathy, severe eccentric left ventricular hypertrophy, decreased contractility with an ejection fraction (EF) <30%, moderate mitral and tricuspid regurgitations, and moderate pulmonary hypertension. Multiple prominent trabeculations were noticed in the middle and apical segments of the left ventricle. The noncompacted to compacted myocardium ratio was >2.5:1. Cardiac catheterization excluded ischemic heart disease. Cardiac magnetic resonance (CMR) imaging confirmed the diagnosis of LVNC. The patient started treatment with carvedilol, ramipril, verospiron, torasemide, and rivaroxaban. An implantable cardioverter-defibrillator (ICD) was recommended. In conclusion, the diagnosis of LVNC in the adult population is often delayed because of similarities with more frequently diagnosed diseases. TTE is the initial diagnostic test of choice. Additional imaging modalities (contrast echocardiography, CMR) can help confirm the diagnosis. Early diagnosis is crucial because of the high incidence of life-threatening complications related to heart failure, thromboembolic events, and ventricular arrhythmias. Additional prospective studies are needed to improve the management and outcomes of this rare cardiomyopathy.

## Introduction

Isolated left ventricular noncompaction cardiomyopathy (LVNC), also known as spongy myocardium, is an extremely rare congenital disorder belonging to unclassified cardiomyopathies by the World Health Organization [1] and classified as a genetic cardiomyopathy by the American Heart Association [2]. Adult prevalence is 0.017-0.26% in observational echocardiographic studies (with a 3:1 male:female ratio) [3]. The disease occurs due to intrauterine arrest of normal myocardial compaction at the fifth to eighth week of embryogenesis resulting in prominent trabeculae, deep intertrabecular recesses, and thickening of the myocardium in two distinct layers: compacted and noncompacted [4]. 

Among the different gene mutations, sarcomere gene mutations are the most common (82%): 71% of MYH7, MYBPC3, and TTN mutations; 11% of ACTC1, ACTN2, MYL2, TNNC1, TNNT2, and TPM1 mutations [5]. Non-sarcomere gene mutations include DES, DSP, FKTN, HCN4, KCNQ1, LAMP2, LMNA, MIB1, NOTCH1, PLN, RYR2, SCN5A, and TAZ [5]. Gene mutations are more frequent in children (44%) compared with adults (30%) [5].

The atypical structure of the myocardium leads to left ventricular dysfunction and dilation, followed by heart failure (HF) symptoms. LVNC causes life-threatening complications related to HF, thromboembolic events, and ventricular arrhythmias. Reported mortality from case series is high, ranging from 35 to 47% over a 42- to 72-month follow-up period from diagnosis [6]; thus, early diagnosis and treatment are imperative. This disease is subdiagnosed, as knowledge regarding proper diagnosis, morbidity, and prognosis is limited. We are reporting a case of isolated LVNC in a 62-year-old male who was referred to our clinic for arrhythmia evaluation. Our aim is to highlight a diagnostic approach to a rare unclassified cardiomyopathy and to stress specific diagnostic signs that make the disease more recognizable.

## Case presentation

**Figure 1:**
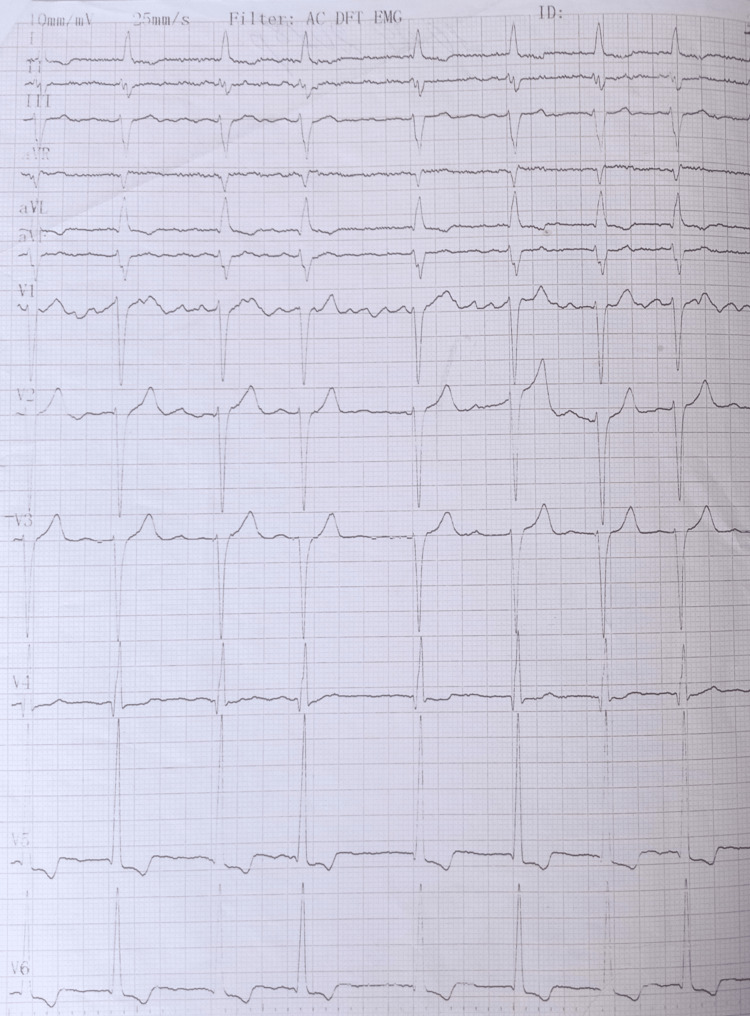
ECG demonstrated atrial fibrillation and ST-T segment depression in the V5-V6 leads. ECG: electrocardiogram.

A 62-year-old white male, without any significant past medical history, no risk factors, and a noncontributory family history, was referred to our clinic for arrhythmia evaluation. The patient had several brief episodes of palpitations and shortness of breath on moderate physical exertion for the past two months. The patient's vital signs were blood pressure (BP)-120/85 mmHg and heart rate (HR)-78 bpm. On physical examination, the apical impulse was displaced to the left, a blowing systolic murmur at the apex with radiation to the left axilla was revealed, and an irregularly irregular rhythm was noticed. The 12-lead ECG demonstrated atrial fibrillation and ST-T segment depression in the V5-V6 leads (Figure 1). 

**Figure 2:**
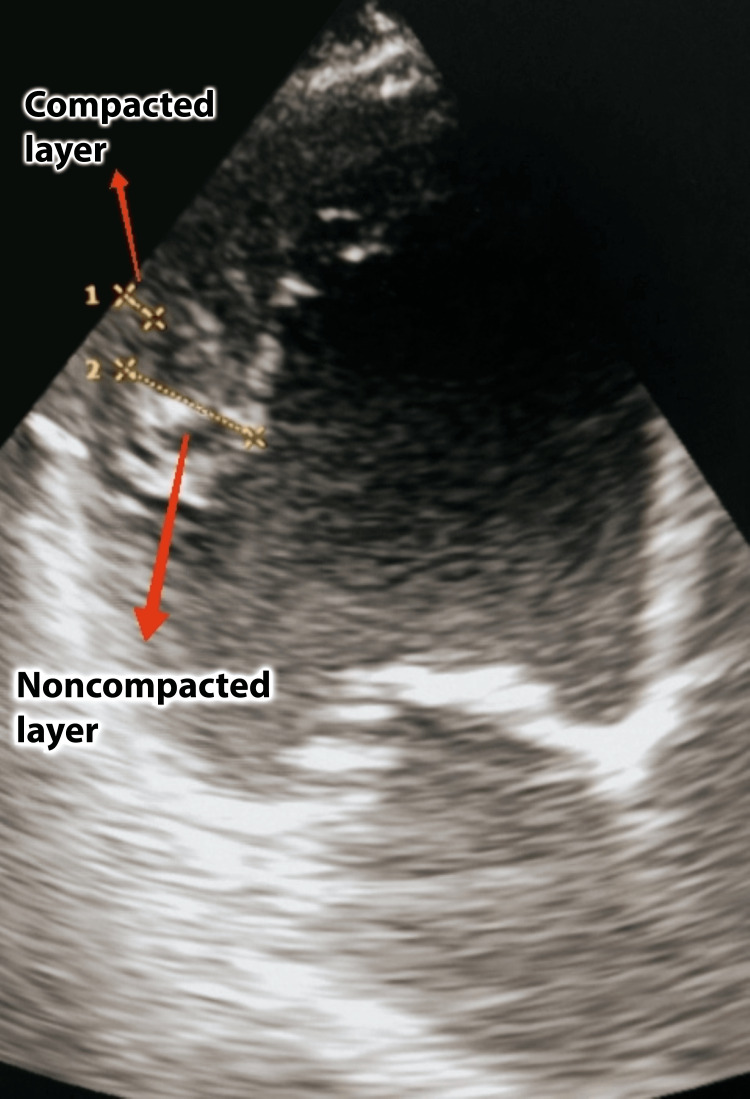
Echocardiography, apical four-chamber view. Multiple trabeculations in the middle and apical segments of the left ventricle. A NC/C in the midventricular segments >2.5:1. NC/C: noncompacted to compacted myocardium ratio. 1 = 0.7 cm; 2 = 1.8 cm.

Transthoracic echocardiogram (TTE) demonstrated signs of dilated cardiomyopathy with enlarged left cardiac chambers: left ventricle (LV), 73 mm; left atrium (LA), 55 mm; severely abnormal LA volume index, 82 mL/m^2^; severely increased myocardial mass index, 164 g/m^2^; and severe eccentric left ventricular hypertrophy. Moderate mitral regurgitation due to leaflet restriction and mild tricuspid regurgitation were revealed. LV contractility was significantly decreased (ejection fraction (EF) <30%). Multiple prominent trabeculations were noticed in the middle and apical segments of the lateral and posterior walls of the left ventricle. The noncompacted to compacted myocardium ratio (NC/C) in the midventricular segments was >2.5:1 in both parasternal short axis and apical four-chamber views (Figure 2). During color Doppler flow imaging, there was direct blood flow from the ventricular cavity into deep intertrabecular recesses. Mild pericardial layer separation (4 mm) was noticed. There were signs suggestive of mild pulmonary hypertension present (tricuspid regurgitation gradient: 33 mmHg).

**Figure 3:**
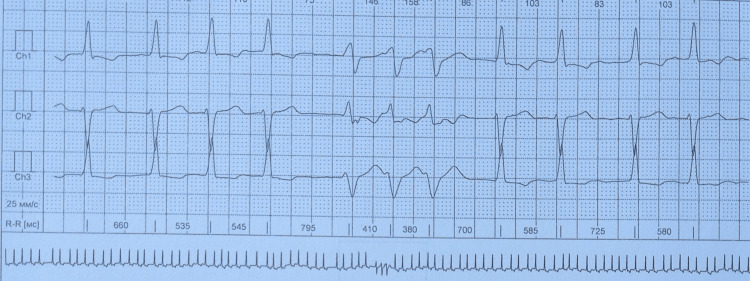
A 24-hour Holter monitor revealed a triplet with a wide QRS complex.

Taking into consideration the patient’s complaints and his age, ischemic heart disease was included in the differential diagnosis. Troponin I levels were negative. Cardiac catheterization was performed, and ischemic heart disease was excluded. A 24-hour Holter monitor showed couplets of premature ventricular contractions, one triplet with a wide QRS complex (Figure 3).

**Figure 4:**
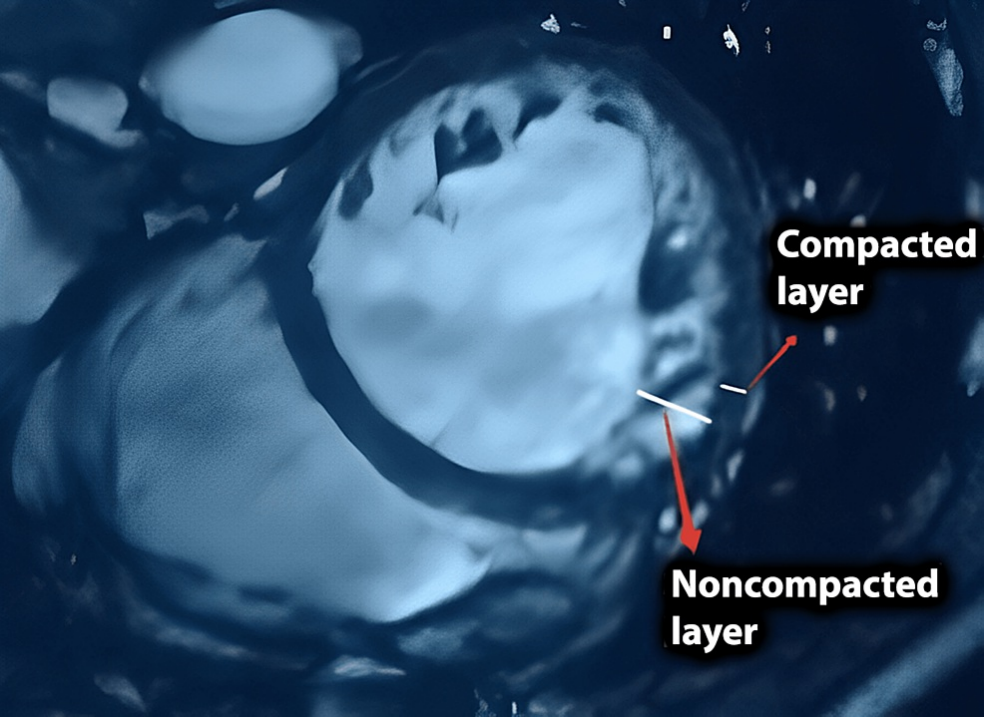
CMR imaging, short-axis view. Bilayered myocardium with a noncompacted to compacted ratio >2.3:1. CMR: cardiac magnetic resonance.

**Figure 5:**
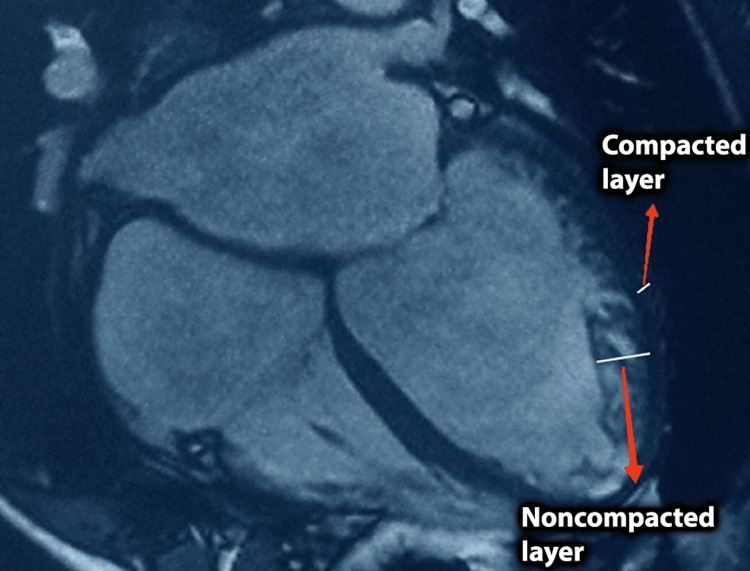
CMR imaging, four-chamber view. Bilayered myocardium with a noncompacted to compacted ratio >2.3:1. CMR: cardiac magnetic resonance.

Cardiac magnetic resonance (CMR) imaging revealed characteristics suggestive of noncompaction cardiomyopathy (bilayered myocardium with a NC/C >2.3:1), confirming the diagnosis (Figures 4, 5).

The patient was prescribed heart failure therapy, including beta-blocker carvedilol, angiotensin-converting enzyme inhibitor (ACEi) ramipril, diuretics verospiron and torasemide, as well as anticoagulant rivaroxaban. In several months, ACEi was switched to sacubitril/valsartan, which was gradually adjusted to the maximum tolerated dosage. The patient had improved clinically, and left ventricular ejection fraction (LVEF) increased to 35%. The patient's two sons underwent cardiological family screening with electrocardiography and echocardiography, which did not reveal any pathology. The patient was followed up every six months for five years with periodic worsening of heart failure, which was corrected with pharmacological adjustments.

## Discussion

The diagnosis of LVNC in an adult population is often delayed or missed because of similarities with more frequently diagnosed diseases, such as ischemic and nonischemic dilated cardiomyopathy, hypertensive heart disease, myocarditis, apical thrombi, and apical hypertrophic and restrictive cardiomyopathy. Physiological trabeculations, false tendons, and aberrant chords may also imitate LVNC. Other diseases mimicking LVNC must be considered as a differential diagnosis, including eosinophilic heart disease, endomyocardial fibroelastosis, cardiac fibroma, cardiac metastases, and intramyocardial abscesses [7].

Clinical presentation of the disease is highly variable. There are nine clinical types of LVNC, including (1) the isolated or benign form with normal systolic function, (2) the arrhythmogenic form, (3) the dilated form, (4) the hypertrophic form, (5) the mixed form, (6) the restrictive form, (7) the biventricular form, (8) the right ventricular hypertrabeculation with normal left ventricle form, and (9) the congenital heart disease form of LVNC [8]. As we know from previously published case reports, the patients may present with acute coronary syndrome, transient ischemic attack (TIA) or stroke, newly diagnosed HF, syncope or presyncope, arrhythmias, including recent-onset paroxysmal atrial fibrillation or newly diagnosed left bundle branch block. Some patients remain asymptomatic at the time of diagnosis but may have a murmur on physical examination [3-8].

Two-dimensional echocardiography is the standard diagnostic test of choice. It can detect a two-layer myocardium in the mid-ventricular and apical levels with a thin compacted layer and a thick noncompacted layer, which consists of trabeculations with deep intertrabecular recesses [9]. There are three sets of echocardiographic criteria for LVNC diagnosis. The Jenni criteria, also known as Zurich criteria, are the most widely accepted validated echocardiographic criteria for LVNC. They are assessed in the parasternal short-axis view at the base, mid, and apical levels [9]. The Jenni criteria include (1) bilayered myocardium with thin compacted and thick noncompacted layers; (2) end-systolic noncompacted to compacted ratio more than 2 (in parasternal short-axis view at mid-ventricular level); (3) communication with the intertrabecular space demonstrated with color Doppler; and (4) absence of coexisting heart disease [9]. The presence of all four criteria is required for the diagnosis of LVNC. In addition, hypokinesis of noncompacted segments may also be present [9].

Alternatively, some clinicians use the Chin or Stöllberger criteria, which have also been validated. The Chin, or California, criterion defines LVNC as the ratio X-to-Y less than or equal to 0.5, where X is the distance from the epicardium to the trough of a trabeculation and Y is the distance from the epicardium to the peak of a trabeculation [10]. This criterion is applied to trabeculae at the LV apex on subxiphoid or apical four-chamber views at end diastole [10]. The Stöllberger criteria, also known as Vienna criteria, emphasize hypertrabeculation. They include (1) more than three prominent trabeculations protruding into the LV cavity, visible in one imaging plane; (2) intertrabecular spaces perfused from the ventricular cavity, visualized on color Doppler imaging [11]. A role for three-dimensional echocardiography has not been established. A role for contrast echocardiography has been incompletely evaluated in LVNC.

CMR imaging helps identify LVNC more precisely. CMR criteria by Peterson with sensitivity of 86% and specificity of 99% include assessment of the noncompacted to compacted ratio, which has to be more than 2.3 during the diastole in any long-axis LV image to make a diagnosis of LVNC [12]. Jacquier et al. proposed to use a highly sensitive and specific criterion: a trabeculated left ventricular mass should be more than 20% of the total LV mass [13]. Cardiac magnetic resonance also identifies the extent of cardiac fibrosis using late gadolinium enhancement, which is related to adverse clinical events [12]. 

Cardiac catheterization is needed to rule out coronary artery disease in an adult population. Cardiological family screening with electrocardiography and echocardiography is important to identify LVNC in family members. Genetic testing may provide unique information to research the genetic heterogeneity of the disease and the genotype‐phenotype correlation.

The incidence of life-threatening complications is high. They are related to HF, ventricular arrhythmias, and thromboembolic events. According to the German LV noncompaction (LVNC) registry, 61% of the patients experience HF symptoms at the time of initial diagnosis [14]. The severity of the symptoms varies from mild to severe, leading to the necessity of LV assist device implantation and heart transplantation. Systolic dysfunction in combination with fibrosis often causes cardiac arrhythmias; 26% of the patients present with arrhythmias as an initial symptom [14]. The range of arrhythmias varies, including both supraventricular and ventricular. The most frequent arrhythmia is atrial fibrillation, which accounts for 18% and often occurs in patients with severely reduced LV function [8,14]. So, Holter monitoring is indicated in patients with LVNC. Thromboembolic complications are related to LV systolic dysfunction and stasis of blood in the intertrabecular recesses and are associated with atrial fibrillation. The incidence of thromboembolic complications ranges from 5% to 38% [15], of which 10-15% lead to neurologic complications [16].

Decreased LV contractility <50%, in particular in patients with midbasal extent of noncompaction, is a statistically significant predictor of overall mortality [17]. HF, graded New York Heart Association, Functional Class III-IV (NYHA III-IV), LVEF <35%, sustained ventricular arrhythmias, and dilated left atrium (left atrial volume index (LAVI) >34 cm^2^/m^2^) are associated with cardiovascular death [16]. It is crucial to diagnose LVNC early, so life expectancy can be extended through early treatment of HF, use of oral anticoagulants (in patients with EF <40% and thromboembolic episodes of atrial fibrillation), use of antiarrhythmic medications, cardiac resynchronization therapy (in patients with low EF and a prolonged QRS duration), automatic ICD implantation (in patients with sustained ventricular tachycardia (VT) or ventricular fibrillation for sudden cardiac death prevention), and heart transplantation when other treatment options are ineffective [2,5,14].

Twenty-three previously published cases of noncompaction cardiomyopathy reported for the past 10 years (2012-2022) in the patient population aged 60 years and older are presented in Table 1. We summarized data to present a comprehensive review, including information about the first authors of the studies, year of publication, patient's age and sex, initial presentation, imaging studies, and patient follow-up. Additional prospective studies are needed to improve the management and outcomes of this rare cardiomyopathy.

**Table 1:** Twenty-three previously published cases of noncompaction cardiomyopathy reported for the past 10 years (2012-2022) in the patient population aged 60 years and older are presented. We summarized data to present a comprehensive review, including information about the first authors of the studies, year of publication, patient's age and sex, initial presentation, imaging studies, and patient follow-up. LVNC: left ventricular noncompaction cardiomyopathy; NC/C: noncompacted to compacted myocardium ratio; TTE: transthoracic echocardiogram; MRI: magnetic resonance imaging; LV: left ventricle; LVEF: left ventricular ejection fraction; ICD: implantable cardioverter-defibrillator; VT: ventricular tachycardia; CT: computed tomography; RV: right ventricle; VSD: ventricular septal defect; TIA: transient ischemic attack; HF: heart failure; MI: myocardial infarction; CXR: chest X-ray; CRT‑D: cardiac resynchronization therapy defibrillator; TEE: transesophageal echocardiogram; 3D TTE: three-dimensional transthoracic echocardiogram; CABG: coronary artery bypass graft.

	Reference, year	Patient age (years), sex	Initial presentation	Imaging studies to diagnose LVNC	Localization, NC/C ratio	Patient follow-up
1	Chien et al. [18], 2022	70 M	Presented with acute coronary syndrome	TTE, cardiac MRI: trabeculations in the LV with apical hypokinesis, LVEF-31%. Coronary angiogram: coronary artery-to-LV fistulas	LV, apex NC/C = 2.4:1	Follow-up in three months: LVEF-30%. The patient was scheduled for ICD implantation
2	Huang et al. [19], 2022	61 F	Presented with HF symptoms and palpitations. ICD implantation due to VT	TTE, CT, ventricular angiography: an isolated spongy RV with prominent and excessive trabeculation of the RV, normal LVEF	RV NC/C = 2.7:1	Follow-up in six months: a stable condition. Missense mutation of TTN gene was revealed
3	Afify et al. [20], 2021	78 M	Presented with symptoms of volume overload after missing hemodialysis session	TTE: Doppler pattern was suggestive of either canalization of the apical mass or hypertrabeculation. Cardiac MRI: trabeculations in the LV apex and apical lateral segment.	LV apex and apical lateral segment	Anticoagulation was started to reduce the thromboembolic risk associated with LVNC
4	Elnazeir et al. [21], 2021	63 M	Admitted with acute bilateral vision loss and dysarthria	TTE: LV dilation with LVEF of 29%. Cardiac MRI: confirmed the presence of LVNC	Apex and left ventricular lateral wall NC/C > 2.3:1	Follow-up in three months after initiating medical management: unremarkable stress test, improved LVEF to 40%
5	Montanarella et al. [22], 2021	66 M	Presented with substernal and left-sided chest pain	TTE: markedly enlarged RV with diffuse trabeculations and hypokinesis, LVEF of 50-55%, s/p surgical repair of VSD. Cardiac MRI: heavily noncompacted RV witha thickened appearance up to 3.4 cm	RV apex	The patent was optimized medically and discharged in stable condition
6	Chen et al. [23], 2020	85 M	Presented with chest pain	TTE: regional noncompaction and associated hypokinesis. Coronary angiography: revealed multiple coronary-cameral fistulas	Anterolateral noncompaction of the LV. NC/C > 2:1	Follow-up visit: condition improved after treatment for acute coronary syndrome
7	Rani et al. [24], 2020	65 M	Admitted for weakness of right upper extremity, HF symptoms, history of polycystic kidney disease	TTE: prominent trabeculations and deep recesses in the LV, LVEF 15–20%. Cardiac MRI: thinned LV myocardium with trabeculations	LV apex and mid cavity NC/C > 3.1:1 (TTE) NC/C > 3.43:1 (MRI)	The patient has been followed up regularly; his condition improved on pharmacotherapy
8	Bath et al. [25], 2019	69 M	Presented with atrial fibrillation complicated with cardiac arrest	TTE: features of LVNC, thrombus in the apical part, LVEF of 10-15%	LV apex and lateral wall NC/C = 2:1	
9	Parekh et al. [26], 2018	61 M	Presented with HF symptoms	TTE: prominent hypertrabeculations in the lateral and apical walls, LVEF of 20-25%. Cardiac MRI: trabeculations at the LV apex	LV apex and lateral wall NC/C = 5:1	Patient exhibited multiple episodes of nonsustained VT and was discharged home with an implanted ICD
10	Pi et al. [27], 2018	78 M	Presented with HF symptoms and palpitations (atrial fibrillation)	Contrast TTE: prominent trabeculations in the lateral-apical wall of the LV, deep intertrabecular recesses filled with slow blood flow	LV apex and lateral wall NC/C > 2:1	Patient was discharged without remarkable adverse events
11	Subahi et al. [28], 2017	69 M	Presented with a stroke for the second time	TTE: increased apical trabeculation of the LV, LVEF of 55%. Cardiac MRI: two-layered appearance of the LV apical chamber, extensive trabecular network	LV, apex NC/C = 2.3:1	Follow-up for 12 months: a stable condition
12	Özyılmaz et al. [29], 2016	62 F	Systolic ejection murmur was revealed on physical examination	TTE: septal hypertrophy with a wall thickness of 21 mm. Cine and contrast-enhanced cardiac MRI: prominent trabeculations.	Apex and left ventricular lateral wall, NC/C = 3:1	
13	Pulignano et al. [30], 2015	67 F	Admitted due to TIA, dyspnea, peripheral edema, associated with recent-onset paroxysmal atrial fibrillation	TTE: markedly dilated LV with hypertrabeculations, severely reduced LVEF of 22%, and an apical thrombus	Apex and inferolateral segments of the LV, NC/C ratio = 2:1	In 12 months, due to anticoagulant discontinuation, the patient presented with HF. TTE: massive LV thrombosis, which caused MI, LVEF = 15%. Sepsis and multiorgan failure lead to death
14	Tanaka et al. [31], 2014	74 F	Referred with dyspnea for three months.	CXR: cardiomegaly TTE: hypertrabeculations and deep intertrabecular recesses. Contrast echocardiography: a direct passage between the LV cavity and deep intertrabecular recesses, LVEF-42%	LV, posterior and lateral wall. NC/C = 3:1	Underwent aortic valve replacement for aortic regurgitation. Follow-up for two years: symptoms of HF improved with LVEF of 64%
15	Dąbrowski et al. [32], 2013	67 M	Presented with newly diagnosed HF and left bundle branch block	TTE: global hypokinesis and LVEF < 30%, increased trabeculation with muscle recesses	Apex, middle inferolateral segments, septum of the LV. NC/C = 2.3-2.4:1	Cardiac resynchronization therapy defibrillator (CRT‑D) was implanted. Follow-up in 32 months: good response to CRT-D treatment
16	Cevik et al. [33], 2012	90 M	Presented with HF for the first time	TTE: trabeculated, sponge-like appearance of the LV	Apical and inferolateral segments of the LV	Follow-up in three months after beginning medical management: remained asymptomatic
17	Parameswaran et al. [34], 2012	80 M	Referred for MRI due to low EF on TTE	Prominent, deep trabeculae arising from the interventricular septum, LVEF-38%	Interventricular septum, anterior, apical, and distal inferior walls of the LV	
18	Toufan et al. [35], 2012	73 M	Admitted with acute MI	TTE: apical LV hypertrabeculations, LVEF-35%	Apex of the LV	The patient underwent CABG and mitral valve repair
19	Cevik et al. [36], 2012	62 F	Presented with HF symptoms	CXR: cardiomegaly. TTE and TEE: hypertrabeculated, sponge-like myocardium, multiple thrombi, LVEF-20%	Apical, inferior apical, and septal segments of the LV	The patient passed away of multiorgan failure
20	Hutchins et al. [37], 2012	63 F	Presented with HF symptoms and h/o small VSD	TTE and TEE: a bilayered myocardium, LVEF = 15 - 20%	Lateral wall of the LV, NC/C=3:1	Conservative treatment was successful
21	Rokutanda et al. [38], 2012	87 M	Presented with HF symptoms	TTE and 3D TTE: LV noncompaction and hypokinesis	Apex of the LV, distance from the epicardial surface to the recesses and to the peak of trabeculation was 0.27	The patient improved after HF treatment, trabeculae converged during systole
22	Steger et al. [39], 2012	61 M	The patient with history of MI, CABG, CRT-D implantation, presented with cardiac decompensation	Autopsy	Tight ventricle with hypertrabeculation	The patient passed away two weeks after a LV assist device implantation due to multiorgan failure
23	Lee et al. [40], 2012	72 M	Presented with HF symptoms	TTE, CT, MRI: thickening of the LV wall with prominent trabeculations and deep intertrabecular recesses, EF-34%	LV cavity NC/C >2:1	The patient improved after HF treatment (LVEF: 40%)

## Conclusions

The diagnosis of LVNC in the adult population is often delayed because of similarities with more frequently diagnosed diseases, such as ischemic and nonischemic dilated cardiomyopathy, apical hypertrophic cardiomyopathy, and hypertensive heart disease. Two-dimensional echocardiography is the standard diagnostic test of choice. Using strict Jenni, Chin, and Stöllberger echocardiographic criteria, we can increase recognition of this disease. Additional imaging modalities (contrast echocardiography, CMR) can help confirm the diagnosis. Early detection and treatment of the disease are crucial to prevent life-threatening complications. Life expectancy can be extended through early treatment of heart failure, use of oral anticoagulants, and automatic ICD implantation. Heart transplantation is the last resort treatment for such patients. Definitive screening for family members should be established. Additional prospective studies are needed to improve the management and outcomes of this rare cardiomyopathy.
